# Experimental model of small-for-size syndrome in pigs

**DOI:** 10.1590/0102-672020260000012e1941

**Published:** 2026-08-07

**Authors:** Paola Sofia Espinoza ALVAREZ, Jhosimar Alvarez DE LÁ HOZ, Daniel Reis WAISBERG, Alessandro Rodrigo BELON, Larissa Barbosa LIMA, Beatriz de Lima SOARES, Ricardo Luis Santamaria GONZALEZ, Rubens Macedo ARANTES, João Paulo Costa dos SANTOS, Pedro Carvalho CASSINO, Cinthia Lanchotte FERREIRA, Marcia Saldanha KUBRUSLY, Luciana Severo BRANDÃO, Vinicius ROCHA-SANTOS, Liliana DUCATTI, Rafael Soares PINHEIRO, Pedro Marin CASTRO, Rodrigo Bronze de MARTINO, Luciana Bertocco HADDAD, Lucas Souto NACIF, Flavio Henrique Ferreira GALVÃO, Luiz Augusto Carneiro D’ALBUQUERQUE, Wellington ANDRAUS

**Affiliations:** 1 Universidade de São Paulo, Faculty of Medicine, Medical Research Laboratory (LIM 37) - São Paulo (SP), Brazil.; 2 Universidade de São Paulo, Faculty of Medicine, Department of Gastroenterology - São Paulo (SP), Brazil.

**Keywords:** Liver transplantation, Organ size, Portal pressure, Liver regeneration, Interleukins, Transplante de fígado, Tamanho do órgão, Pressão na veia porta, Regeneração hepática, Interleucinas

## Abstract

**Background:**

Small-for-size syndrome (SFSS) is a serious complication after partial liver transplantation or extended hepatectomy. Despite advances, the syndrome remains a complex entity with a multifactorial pathophysiology and a lack of standardized therapeutic approaches.

**Aims:**

This study aims to establish a translational model of porcine SFSS, integrating hemodynamic, histopathological, and molecular analyses to quantify the tissue expression of biomarkers to assess liver regeneration.

**Methods:**

Ten liver transplants were performed: eight with small (partial) for size (donors underwent a 70% hepatectomy) and two with whole liver. The recipients were followed until death (euthanasia due to severe morbidity) or for five days. Hemodynamic data, laboratory analyses, and liver tissue biopsies were collected. Immunohistochemistry was performed to assess the expression of TNF-α, IL-1β, NF-κB, and VEGF. Gene expression levels of VEGF, IL-6, IL-10, and NF-κB were quantified using reverse transcription polymerase chain reaction.

**Results:**

In the partial group, survival rates were 75% in the immediate postoperative period, 25% at 48 hours, and 12.5% at five days. Immunohistochemistry showed a significant increase in TNF-α and NF-κB proteins after reperfusion and at the end of surgery, respectively. Gene expression analysis showed a significant increase in IL-6 and IL-10 at the end of surgery.

**Conclusions:**

This study represents a methodological advance by performing the first integrated evaluation of protein and gene expression (TNF-α, IL-6, IL-1, IL-10, VEGF, and NF-κB) correlated with hemodynamic, histopathological, and functional parameters in the small-for-size pig model.

## ARTICLE HIGHLIGHTS

• Small-for-size syndrome (SFSS) is a serious complication after partial liver transplantation or extensive liver resections.

• Despite advances, the syndrome remains a complex entity with a multifactorial pathophysiology and a lack of standardized therapeutic approaches.

• Despite the extensive literature on SFSS and liver regeneration, a significant gap remains in the availability of experimental models.

• The analysis of protein expression and gene expression of key mediators in liver regeneration, may elucidate the mechanisms involved in graft dysfunction and recovery in SFSS.

## Summary

**CENTRAL MESSAGE:**

This study centers on small-for-size syndrome, a serious complication when a transplanted liver graft is too small, and uses a pig model to understand the pathophysiology, monitoring survival and analyzing tissue for key proteins and genes linked to inflammation and regeneration.

**PERSPECTIVES:**

This study has practical value because it establishes a reliable animal model to study small-for-size syndrome, identifying specific biological markers linked to inflammation and poor regeneration. These findings provide a foundation for future researchers to test new treatments. Ultimately, this could help make partial liver transplants more successful, allowing more patients to receive life-saving organs despite the shortage of donors.

## VISUAL ABSTRACT

**Figure 1.**
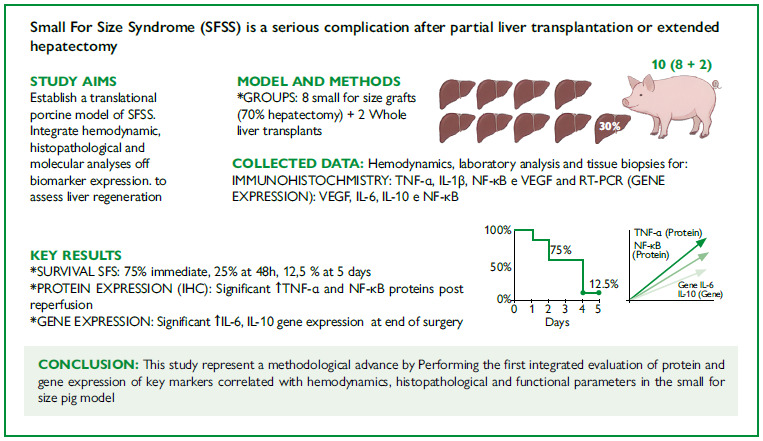

## RESUMO

**Racional:**

A síndrome *small-for-size* (SFSS) do fígado é uma complicação grave após transplante hepático parcial ou hepatectomia extensa. Apesar dos avanços, esta síndrome permanece como uma entidade complexa, com fisiopatologia multifatorial e falta de abordagens terapêuticas padronizadas.

**Objetivos:**

Este estudo visa estabelecer um modelo translacional de SFSS em suínos, integrando análises hemodinâmicas, histopatológicas e moleculares para quantificar a expressão tecidual de biomarcadores e avaliar a regeneração hepática.

**Métodos:**

Dez transplantes hepáticos foram realizados: oito com fígado pequeno (parcial) para o tamanho (doadores submetidos a hepatectomia de 70%) e dois com fígado total. Os receptores foram acompanhados até o óbito (eutanásia em decorrência de morbidade grave) ou por cinco dias. Dados hemodinâmicos, análises laboratoriais e biópsias de tecido hepático foram coletados. A imuno-histoquímica foi realizada para avaliar a expressão de TNF-α, IL-1β, NF-κB e VEGF. Os níveis de expressão gênica de VEGF, IL-6, IL-10 e NF-κB foram quantificados por reação em cadeia da polimerase com transcrição reversa.

**Resultados:**

No grupo parcial, as taxas de sobrevida foram de 75% no período pós-operatório imediato, 25% em 48 horas e 12,5% em cinco dias. A imuno-histoquímica mostrou um aumento significativo nas proteínas TNF-α e NF-κB após a reperfusão e ao final da cirurgia, respectivamente. A análise de expressão gênica mostrou um aumento significativo em IL-6 e IL-10 ao final da cirurgia.

**Conclusões:**

Este estudo representa um avanço metodológico ao realizar a primeira avaliação integrada da expressão proteica e gênica de marcadores-chave (TNF-α, IL-6, IL-1, IL-10, VEGF e NF-κB), correlacionados com parâmetros hemodinâmicos, histopatológicos e funcionais no modelo de porco de pequeno porte.

## INTRODUCTION

Small-for-size syndrome (SFSS) is a serious complication after partial liver transplantation or extensive liver resections, characterized by liver dysfunction with prolonged hyperbilirubinemia, coagulopathy, refractory ascites, and encephalopathy. Its pathophysiology involves a complex interaction between hemodynamic and inflammatory factors and alterations in hepatic microarchitecture, resulting in failure of organ regeneration^14^.

Kiuchi et al.^25^ established that grafts with a graft-to-recipient weight ratio (GRWR) <0.8% or a standard liver volume <30-40% are associated with a higher risk of SFSS, and that other factors such as hepatic steatosis, pre-existing portal hypertension, and ischemia-reperfusion injury aggravate the syndrome^9^ ^,^ ^25^.

Reduced liver volume leads to increased portal flow, causing shear stress in sinusoidal endothelial cells, resulting in microvascular injury, activation of inflammatory cytokines such as tumor necrosis factor alpha (TNF-α), interleukin-6 (IL-6), interleukin-1 beta (IL-1β), and impaired regeneration. This process is mediated by pathways such as nuclear factor kappa B (NF-κB) and signal transducer and activator of transcription 3 (STAT3), while angiogenic vascular endothelial growth factor (VEGF) and anti-inflammatory iterleukin-10 (IL-10) factors modulate the response. Proper liver regeneration requires a balance between hepatocellular proliferation and vascular remodeling, which is frequently compromised in SFSS^12^ ^,^ ^29^ ^,^ ^34^ ^,^ ^35^ ^,^ ^50^.

Animal models, particularly pigs, have been essential for studying SFSS due to their anatomical and physiological similarities to humans. However, challenges such as hemodynamic instability and postoperative management limit their application^3^ ^,^ ^8^ ^,^ ^10^ ^,^ ^11^ ^,^ ^15^ ^,^ ^16^ ^,^ ^19^ ^,^ ^32^.

This study proposes an experimental SFSS model of liver transplantation to evaluate hemodynamic parameters, inflammatory, anti-inflammatory, and regenerative biomolecular markers (TNF-α, IL-1β, IL-6, NF-κB, IL-10, and VEGF), aiming to define their role in impaired regeneration. The results may contribute to defining safe thresholds for partial transplants and identifying therapeutic targets and interventional strategies.

## METHODS

The experiments were performed at the Medical Research Laboratory of the Digestive System Organs Department at Faculty of Medicine of the Universidade de São Paulo.

This study was approved by the Ethics Committee on the Use of Animals (CEUA) of the Faculty of Medicine, Universidade de São Paulo (Protocol No. 2199/2025).

A total of 20 animals were divided into two groups:

Group 1 - Liver transplant with total graft (donors n=2; recipients n=2).

Group 2 - Liver transplant with partial graft (donors n=8; recipients n=8).

Hemodynamic data and biological materials (blood and liver tissue) were collected for laboratory, histological, and genetic analyses.

### Anesthesia

Pigs were fasted for 12 hours and received pre-anesthesia with xylazine (2 mg/kg) and ketamine (10 mg/kg) intramuscularly. Induction was performed with propofol (3-5 mg/kg, intravenous), followed by orotracheal intubation. Anesthesia was maintained with isoflurane (1.5-2.5%) and continuous fentanyl infusion (0.1 µg/kg/min), under volume-controlled mechanical ventilation (10 mL/kg, positive end-expiratory pressure [PEEP]=5 mmHg, fraction of inspired oxygen [FiO_2_]=50%). Monitoring included cardioscopy, oximetry, capnography, and invasive and central venous blood pressure. Catheters were inserted into the jugular veins (Swan-Ganz for hemodynamics; 16 Fr for veno-venous bypass) and carotid/femoral arteries (blood pressure and blood gas analysis).

### Liver transplant with whole graft

The surgery was performed using the method described by Fondevila et al.^12^, with one modification: a veno-venous bypass was employeed, connecting the vena cava and portal vein to the left jugular vein for enhanced hemodynamic control (Figure 1).

**Figure 1.**
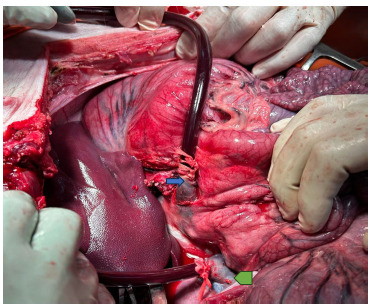
Veno-venous bypass.

Colored arrows indicate: blue: portal vein and green: vena cava.

Source: Author’s own work; 2025.

### Liver transplant with partial graft

To establish the small-for-size model, the liver was reduced by approximately 30% of its total volume, based on a literature review.

A mixed technique of partial hepatectomy was used in the donor and then in the back-table, allowing for less blood loss and adequate control of hemostasis during the implant.

During the donor surgery, steps for liver mobilization, identification, isolation, and repair of the hepatic hilum structures and abdominal aorta were the same.

The vascular pedicles of the left lateral and left medial segments were dissected, ligated, and sectioned. After delimiting the parenchyma, vascular clamps were placed on the left lateral segment, and the parenchyma was sectioned with an electroscalpel, and a continuous suture (4-0 polypropylene suture) was made for vascular control; the same procedure was performed for the left medial segment. After hemostasis was achieved with bipolar cautery, the same steps of the extraction surgery were continued.

**Figure 2.**
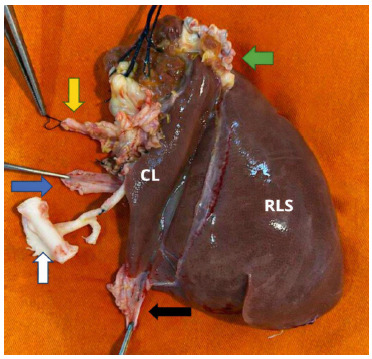
Final appearance of the partial graft.

On the back-table surgery, the vascular pedicle of the right medial segment was ligated and sectioned, followed by parenchymal sectioning using the Kellyclasia technique, ligation of the vascular and biliary structures (3-0 and 4-0 cotton), and hemostasis was controlled with bipolar electrocautery. The vena cava, hepatic artery, and portal vein were prepared according to the standard technique (equivalent to a total graft). Finally, the right lateral segment and the caudate lobe (approximately 30% of the original volume) were preserved for implantation (Figure 2).

**Figure 3.**
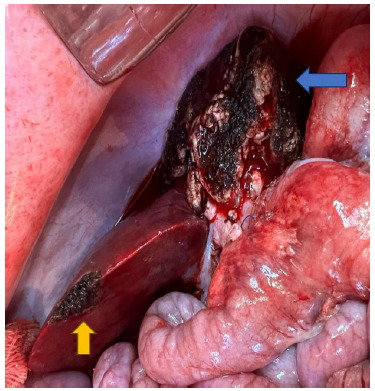
Appearance of the partial graft at the end of the procedure.

The transplant of the partial graft followed the same steps as the whole liver transplant (Figure 3).

Colored arrows indicate: yellow: common bile duct; blue: portal vein; white: abdominal aorta; black: infrahepatic vena cava; green: suprahepatic vena cava.

CL: caudate lobe; RLS: right lateral segment.

Source: Author’s own work; 2025.

Colored arrows indicate: yellow: biopsy area; blue: raw area of hepatectomy.

Source: Author’s own work; 2025.

### Postoperative management

**Figure 4.**
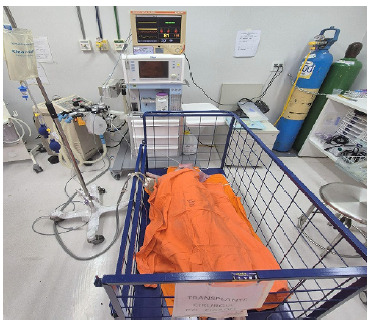
Immediate postoperative monitoring at the LIM37 Medical Research Laboratory.

Source: Author’s own work; 2025.

Stable animals received analgesia (dipyrone+tramadol), antibiotic (ceftriaxone), and immunosuppression (methylprednisolone), with monitoring for five days. Euthanasia was performed on the 5th day (necropsy) or earlier if necessary due to severe morbidity. Disposal was made according to biosafety protocols (Figure 4).

### Blood and serum analysis

Blood sample from the donor was collected at baseline and from the recipient, at baseline and at 1 and 3 hours after portal reperfusion. In these samples, the serum levels of aspartate aminotransferase, alanine aminotransferase, creatinine, alkaline phosphatase, gamma-glutamyl transferase, total bilirubin, direct bilirubin and indirect bilirubin, as well as complete blood count and blood gas, were determined.

### Tissue analysis

Liver tissue fragments were obtained from the donor at the beginning of surgery (T0), during back-table surgery (back), and from the recipient, one hour after portal reperfusion (T3), and at the end of the procedure (T4). Each biopsy was divided into two sections: one preserved in 10% formaldehyde for subsequent paraffin embedding for hematoxylin-eosin-stained slides and for immunohistochemistry; and the second fragment, in liquid nitrogen, for subsequent gene expression analysis.

### Immunohistochemistry

For slide preparation, liver tissue sections were placed on silanized slides. The deparaffinization process was carried out by placing the slides in hot xylene for 15 minutes, followed by two cold xylene baths. Hydration was then conducted in two absolute alcohol baths (95°C and 70°C). Running water was used to retrieve antigenic sites. The recovery was performed at a high temperature of 125°C for one minute using a citrate buffer (pH 6.0). After this period, the slides were washed three times in trisphosphate buffer (pH 7.4). Endogenous peroxidase blocking was achieved with 10-volume hydrogen peroxide (3%) for four 5-minute washes, followed by washing in running water and Tris buffer (pH 7.4). Next, nonspecific protein blocking was performed with an animal-free blocking solution buffer for 10 minutes.

The slides were incubated overnight at 4°C with the following antibodies: TNF-α (1:100), IL-1β (1:100), NF-κB (1:100), and VEGF (1:200).

After incubation, the slides were washed in Tris buffer. They were incubated with the Easylink One secondary antibody for 30 minutes at 37°C. The chromogen diaminobenzidine was used for signaling.

Finally, counterstaining with Harris hematoxylin and mounting with Permount were performed for all reactions.

#### Gene expression analysis

Total RNA extraction was conducted from approximately 100 mg of liver tissue. The protocol began with the addition of TRIzol™ reagent, followed by homogenization of the sample in a TissueLyser using 5 mm aluminum beads for 15 minutes. After homogenization, chloroform was added and the mixture was vortexed. Centrifugation was carried out at 4°C for 15 minutes. This process resulted in the formation of three distinct phases: a lower phenolic phase (proteins), a middle phase (DNA - deoxyribonucleic acid), and an upper aqueous phase (RNA - ribonucleic acid).

The aqueous phase was carefully transferred to a new microcentrifuge tube, and ice-cold isopropyl alcohol was added for RNA precipitation. The samples were incubated at room temperature for 10 minutes and subsequently centrifuged at 4°C for 10 minutes. The supernatant was removed, and the RNA precipitate was washed with 75% ethanol. A further centrifugation was performed at 4°C for 10 minutes. The RNA obtained was solubilized in sterile DNase/RNase-free ultrapure water. Total RNA concentration was determined using a NanoDrop Spectrophotometer, with readings taken at 260 nm and 280 nm. The standard value of 1 optical density (260 nm)=40 µg/mL RNA was used to calculate concentration. RNA purity was assessed by the absorbance ratio of 260/280 nm, and only samples with a ratio ≥1.8 were considered for use. The integrity of the extracted RNA was verified by 1.0% agarose gel electrophoresis to confirm the presence of bands corresponding to 18S and 28S ribosomal RNAs. The extracted RNA was stored at -80°C until use.

The extracted RNA was converted to cDNA (complementary DNA) using the High-Capacity RNA-to-cDNA kit. The reaction was conducted in an Applied Biosystems™ StepOnePlus Thermocycler, following the program: 37°C for 60 minutes and 95°C for 5 minutes. The synthesized cDNAs were diluted (1:5) and stored at −20°C until used in quantitative reverse transcription polymerase chain reaction (qRT-PCR).

Gene expression analysis was performed on a StepOnePlus™ thermocycler using TaqMan^®^ Gene Expression Assays. The qRT-PCR were performed in duplicate. The following target genes were selected: NF-κB, VEGF, IL-6, IL-10, with beta-actin (ACTB) used as the endogenous control.

Normalization was also performed using the ROX (6-carboxy-X-rhodamine) passive reference dye signal to correct for fluctuations in readings due to possible volume variations and evaporation during the reaction. Relative quantification was calculated using the delta-delta comparative threshold (ΔΔCt) method, with the endogenous ACTB gene as data normalizer. The threshold-cycle (Ct) value for each target gene was normalized by subtracting the mean Ct value of the endogenous gene (ΔCt = Ct target gene - Ct control gene).

### Data and statistical analysis

Each quantitative variable was inspected by histograms and Q-Q (quantile-quantile) plots, followed by the Shapiro-Wilk test. Variables with p>0.05 were considered approximately normal, justifying presentation as mean±standard deviation; the others were summarized by median and interquartile range.

## RESULTS

A total of 20 male pigs were used, and 10 liver transplants were performed: two with total grafts and eight with partial grafts. In the total graft group, the median survival time was 61.00 (2.00; 120.00) hours, and in the partial graft group, it was 1.00 (0.50; 26.50) hours. Survival rates in the partial graft group were 75% in the immediate postoperative period, 25% at 48 hours, and 12.5% up to five days.

### Surgical procedure and hemodynamic parameters analysis

The median cold ischemia time in the total and partial graft groups was 195.50 (163.00; 228.00) and 204.00 (188.00; 214.00) minutes, respectively. The warm ischemia time was 36.50 (33.00; 40.00) minutes in the total graft group and 32.00 (29.00; 34.50) minutes in the partial graft group (Table 1)

**Table 1.** Sample and surgical procedure characteristics.

Variable	Whole transplant n=2*	Partial transplant n=8*
Donor weight (kg)	40.50 (31.00-50.00)	30.25 (20.50-32.35)
Recipient weight (kg)	36.50 (27.00-46.00)	30.25 (24.55-34.35)
Graft weight (kg)	1.03 (0.92-1.14)	0.81 (0.67-0.84)
Weight after reduction^†^	0.00 (0.00-0.00)	0.22 (0.19-0.29)
GRWR (%)	2.94 (2.48-3.40)	0.79 (0.73-0.90)
Cold ischemia (min)	195.50 (163.00-228.00)	204.00 (188.00-214.00)
Warm ischemia (min)	36.50 (33.00-40.00)	32.00 (29.00-34.50)
Survival (h)	61.00 (2.00-120.00)	1.00 (0.50-26.50)

*n (%); ^†^Weight reduced only for partial transplant.

Median: interquartile range; GRWR: graft weight/recipient weight ratio.

**Table 2.** Hemodynamic data: partial group.

Variable	T0 n=8*	T3 n=7*	T4 n=6*
PAP (mmHg)	17 (5); 17 (13-23)	20 (3); 20 (17-22)	27 (17); 19 (15-47)
PAOP (mmHg)	9.57 (3.10); 9.00 (7.00-12.00)	8.50 (0.71); 8.50 (8.00-9.00)	7.00 (NA); 7.00 (7.00-7.00)
CO (L/min)	10.12 (2.08); 10.54 (8.47-11.84)	6.13 (2.07); 6.30 (5.99-7.80)	7.15 (2.84); 7.04 (5.14-9.17)
SBP (mmHg)	96 (15); 97 (90-105)	64 (20); 56 (49-83)	71 (16); 69 (67-80)
DBP (mmHg)	64 (18); 65 (51-75)	35 (11); 31 (28-47)	33 (9); 30 (26-43)
MAP (mmHg)	76 (18); 81 (64-85)	46 (13); 43 (38-56)	45 (8); 43 (40-53)
HR (bpm)	103 (14); 102 (90-114)	141 (36); 114 (113-184)	166 (42); 176 (130-195)
PVP (mmHg)	7.1 (2.4); 6.5 (5.5-9.0)	14.2 (3.6); 13.0 (11.0-18.0)	

*Mean: standard deviation; Median: interquartile range.

T0: beginning of surgery; T3: post-revascularization; T4: end of surgery; PAP: pulmonary artery pressure; PAOP: pulmonary artery occlusion pressure; CO: cardiac output; SBP: systolic blood pressure; DBP: diastolic blood pressure; MAP: mean arterial pressure; HR: heart rate; PVP: portal vein pressure.

### Laboratory analysis

The mean hemoglobin in the partial graft group was 7.6±3.6 g/dL, lower than that in the total graft group, which was 10.67±1.44 g/dL. Among the liver function tests, there was an important increase in aspartate aminotransferase, with a mean of 237±322 U/L, compared to the total graft group, which had a mean of 92±107 U/L. Total bilirubin remained normal in the partial graft group, at 0.96±0.81 mg/dL, and slightly increased in the total graft group, at 1.15±0.038 mg/dL. Creatinine values remained normal in both groups (Table 3).

**Table 3.** Recipient blood count and biochemistry data.

Variable	Total*	Partial*
Hemoglobin (g/dL)	10.67 (1.44); 10.10 (9.60-12.30)	7.6 (3.6); 8.9 (8.3-9.7)
Hematocrit (%)	34.40 (5.46); 31.30 (31.20-40.70)	25 (12); 29 (28-31)
Leukocytes (×10^3^/mm^3^)	12.13 (2.68); 11.70 (9.70-15.00)	18 (13); 16 (15-17)
Platelets (×10^3^/mm^3^)	278 (127); 248 (169-417)	236 (156); 235 (119-348)
ALT (U/L)	28 (17); 27 (17-41)	32 (21); 30 (22-37)
AST (U/L)	92 (107); 28 (15-164)	237 (322); 137 (22-265)
Creatinine (mg/dL)	0.73 (0.30); 0.83 (0.82-0.88)	0.91 (0.64); 0.77 (0.61-1.18)
Alkaline phosphatase (U/L)	137 (76); 140 (119-197)	266 (266); 207 (123-291)
GGT (U/L)	29 (13); 21 (19-42)	36 (23); 27 (22-51)
Total bilirubin (mg/dL)	1.15 (0.72); 1.12 (0.85-1.81)	0.96 (0.81); 0.85 (0.20-1.49)
Direct bilirubin (mg/dL)	0.111 (0.038); 0.124 (0.089-0.124)	0.10 (0.10); 0.05 (0.02-0.16)
Indirect bilirubin (mg/dL)	1.04 (0.71); 0.96 (0.73-1.68)	0.86 (0.77); 0.73 (0.15-1.43)

*Mean: standard deviation; Median: interquartile range.

ALT: alanine aminotransferase; AST: aspartate aminotransferase; GGT: gamma glutamyl transferase.

### Histological analysis

In the partial graft group, portal endothelial injury was observed at T3 in 75% of the samples and, at T4, in 100% of the samples. There was intense necrosis in 67% of the samples at T4. Periportal hemorrhage was found at T3 in 63% of the samples and, at T4, in 100%. Sinusoidal dilation was moderate in 67% of the samples at T4. Hemorrhage was absent at T0 and at the back-table. At T3, 50% of the samples had moderate hemorrhage, and at T4, 67% had intense hemorrhage. Apoptosis findings were mild in 50% at T3, and severe in 67% at T4. Mild periportal edema was found in 63% 67% of the samples at T3. Microvacuolar changes were mild in 75% of cases at T3 and in 67% at T4 (Table 4).

**Table 4.** Histological analysis.

Variables	Partial group		
	Back n=7 (%)*	T3 n=8 (%)*	T4 n=6 (%)*
Necrosis			
Absent	7 (100)	1 (13)	0 (0)
Mild	0 (0)	2 (25)	1 (17)
Moderate	0 (0)	3 (38)	1 (17)
Intense	0 (0)	2 (25)	4 (67)
Apoptosis			
Absent	7 (100)	1 (13)	0 (0)
Mild	0 (0)	4 (50)	1 (17)
Moderate	0 (0)	1 (13)	1 (17)
Intense	0 (0)	2 (25)	4 (67)
Inflammatory infiltrate			
Absent	6 (86)	4 (50)	1 (17)
Mild	1 (14)	3 (38)	3 (50)
Moderate	0 (0)	1 (13)	1 (17)
Intense	0 (0)	0 (0)	1 (17)
Sinusoidal dilation			
Absent	3 (43)	0 (0)	0 (0)
Mild	4 (57)	4 (50)	0 (0)
Moderate	0 (0)	3 (38)	4 (67)
Intense	0 (0)	1 (13)	2 (33)
Hemorrhage			
Absent	7 (100)	1 (13)	0 (0)
Mild	0 (0)	2 (25)	1 (17)
Moderate	0 (0)	4 (50)	1 (17)
Intense	0 (0)	1 (13)	4 (67)
Periportal edema			
Absent	7 (100)	2 (25)	1 (17)
Mild	0 (0)	5 (63)	2 (33)
Moderate	0 (0)	1 (13)	3 (50)
Intense	0 (0)	0 (0)	0 (0)
Microvacuolar alteration			
Absent	6 (86)	1 (13)	1 (17)
Mild	1 (14)	6 (75)	4 (67)
Moderate	0 (0)	1 (13)	1 (17)
Intense	0 (0)	0 (0)	0 (0)
Portal endothelial injury	1 (14)	6 (75)	6 (100)
Portal hemorrhage	0 (0)	5 (63)	6 (100)

*n (%).

T0: beginning of surgery; Back: tray surgery; T3: after reperfusion; T4: end of procedure.

### Immunohistochemical analysis

In the partial graft group, VEGF maintained mild to moderate expression without significant changes at all surgical time points.

IL-1β expression increased significantly at T4.

TNF-α maintained mild expression, but with a significant increase in mean expression between T0 and T3.

**Figure 5.**
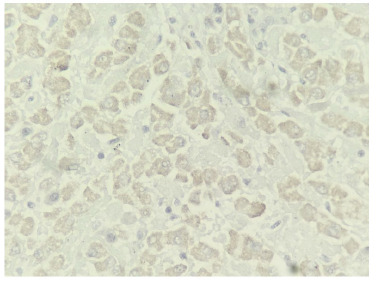
Tumor necrosis factor alpha immunostaining in a liver tissue sample (T4). Mild expression.

Source: Survey data; 2025.

**Figure 6.**
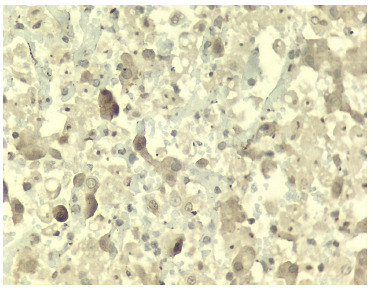
Interleukin-1 beta immunostaining in a liver tissue sample. Moderate expression.

Source: Survey data, 2025.

NF-κB at T0 and back remained mildly expressed, with a significant increase in its expression to moderate at T4 (Tables 5 and 6, Figures 5 and 6).

**Table 5.** Immunohistochemical protein expression.

Variables	Partial group				
	T0 n=8*	Back n=7*	T3 n=8*	T4 n=6*	p-value^†^
VEGF	98-100	98-100	96-100	113-110	0.051
IL-1β	82-93	99-100	96-100	104-105	0.023
TNF-α	23-25	61-70	92-100	81-93	0.006
NF-κB	93-96	90-100	109-110	125-130	0.004

*Mean, Median; ^†^Kruskal-Wallis rank sum test.

T0: beginning of surgery; Back: tray surgery; T3: after reperfusion; T4: end of procedure; VEGF: vascular endothelial growth factor; IL-1β: interleukin-1 beta; TNF-α: tumor necrosis factor alpha; NF-κB: nuclear factor kappa B.

**Table 6.** Post hoc comparisons (Dunn’s test, BH-adjusted p-value) in the partial group.

Variables	T0 vs. Back	T0 vs. T3	T0 vs. T4	Back vs. F3	Back vs. T4	T3 vs. T4
VEGF	0.789	0.725	0.081	0.671	0.116	0.052
IL1-β	0.197	0.191	0.013 *	0.865	0.191	0.191
TNF-α	0.148	0.005^†^	0.034 *	0.176	0.460	0.460
NF-κB	0.566	0.064	0.006^†^	0.193	0.022*	0.269

*p<0,05; ^†^p<0,01 (p-values adjusted by the Benjamini-Hochberg method).

T0: beginning of surgery; Back: tray surgery; T3: after reperfusion; T4: end of procedure; VEGF: vascular endothelial growth factor; IL-1β: interleukin-1 beta; TNF-α: tumor necrosis factor alpha; NF-κB: nuclear factor kappa B.

### Gene expression analysis

The analysis of the expression of the NF-κB and VEGF genes in the different stages of liver transplantation with partial graft did not reveal a statistically significant difference between the four groups corresponding to the surgical times (T0, back, T3, and T4) evaluated by the Kruskal-Wallis test (p=0.134 and p=0.408), respectively.

Analysis of IL-6 gene expression showed a statistically significant difference between the four groups (T0, back, T3, and T4) assessed by the Kruskal-Wallis test (p=0.003, p<0.05). IL-6 expression was significantly higher in the T4 group compared to the T0 group (p=0.03, p<0.05).

Analysis of IL-10 gene expression revealed a statistically significant difference between the four groups (T0, back, T3, and T4) assessed by the Kruskal-Wallis test (p=0.036, p<0.05). IL-10 gene expression was significantly higher in the back-table group compared to the T3 group (p=0.006, p<0.05). There was also a statistically significant difference between the T3 and T4 groups (p=0.045, p<0.05) (Tables 7 and 8).

**Table 7.** Gene expression.

Variables	Partial group				
	T0 n=6*	Back n=6*	T3 n=6*	T4 n=6*	p-value^†^
VEGF	0.38-0.39	0.47-0.46	0.46-0.45	0.66-0.54	0.400
IL-6	0.27-0.33	1.06-0.99	0.29-0.17	1.94-1.84	0.005
IL-10	0.28-0.32	0.68-0.57	0.58-0.28	1.02-0.88	0.036
NF-κB	0.62-0.62	0.82-0.75	1.10-0.98	0.74-0.75	0.130

*Mean, Median; ^†^Kruskal-Wallis rank sum test.

T0: beginning of surgery; Back: tray surgery; T3: after reperfusion; T4: end of procedure; VEGF: vascular endothelial growth factor; IL-6: interleukin-6; IL-10: interleukin-10; NF-κB: nuclear factor kappa B.

**Table 8.** Post hoc comparisons (Dunn’s test, BH-adjusted p-value) in the partial group.

Variables	T0 vs. Back	T0 vs. T3	T0 vs. T4	Back vs. F3	Back vs. T4	T3 vs. T4
VEGF	0.694	0.662	0.615	0.744	0.694	0.744
IL-6	0.062	0.838	0.019*	0.049*	0.585	0.019*
IL-10	0.158	0.462	0.037*	0.393	0.393	0.136
NF-κB	0.406	0.120	0.470	0.406	0.713	0.406

*p<0,05 (p-values adjusted by the Benjamini-Hochberg (BH) method).

T0: beginning of surgery; Back: tray surgery; T3: after reperfusion; T4: end of procedure; VEGF: vascular endothelial growth factor; IL-6: interleukin-6; IL-10: interleukin-10; NF-κB: nuclear factor kappa B.

## DISCUSSION

The shortage of organs for liver transplantation has driven the search for innovative surgical alternatives to reduce long waiting lists and mortality associated with terminal liver disease. One strategy is living donor liver transplantation, performed for the first time in the world in December 1988 at the University of São Paulo, Brazil, by a team led by Dr. Silvano Raia. Although the initial outcome was unfavorable (death on the sixth postoperative day due to renal complications), this case demonstrated the feasibility of the procedure^16^ ^,^ ^28^ ^,^ ^37^.

Living donor liver transplantation represents a significant advance; however, it has complications, which limit its routine use. SFSS is one of them; it has been described since the 1990s and remains a complex entity, with a multifactorial pathophysiology and a yet to be standardized therapeutic approach^5^ ^,^ ^22^ ^,^ ^27^ ^,^ ^34^.

Several research groups have dedicated themselves to studying the syndrome, its pathophysiological mechanisms, and developing therapeutic strategies^34^. Most of these approaches focus on modulating portal flow, which is recognized as a key factor in the syndrome’s genesis. Interventions include splenectomy and/or splenic artery ligation^39^, portosystemic shunts in humans^21^ ^,^ ^42^, validation in a swine model^18^ ^,^ ^43^ ^,^ ^46^, and pharmacological therapy with somatostatin in experimental models^17^ ^,^ ^31^.

Despite these advances, the lack of consensus on the ideal approach reflects the complexity of SFSS and the need for further studies. Since the 1980s, several swine models have emerged for the study of the syndrome, establishing themselves as a fundamental tool for understanding this complex clinical entity. These models, which offer unique advantages, allow the recreation of the pathophysiology of the disease, the investigation of molecular mechanisms, and the testing of surgical and pharmacological interventions before clinical application. Two main experimental approaches have been employed: extended hepatectomies and liver transplants with reduced grafts^2^ ^,^ ^7^ ^,^ ^13^ ^,^ ^24^ ^,^ ^31^. This study used a graft corresponding to the right lateral segment and caudate lobe of the liver, obtaining a median GRWR of 0.79% (0.73; 0.90). The immediate postoperative survival rate was 75%, decreasing to 25% in the first 48 hours. These findings are in line with the data of Hori et al.^20^, who reported a survival rate of 20% at 48 hours. In the first 40 cases performed by the group, Hessheimer et al. reported survival rates of 35% at 48 hours^18^. These variations between different centers could be related to the learning curve of the technique, postoperative management protocols, and animal selection criteria. Our hemodynamic data demonstrated baseline portal vein pressure values of 7.1±2.4 mmHg and 14.2±3.6 mmHg after graft reperfusion, very similar to those found in the study by Asakura et al.^1^, who, using portocaval shunt, demonstrated a significant improvement in survival through the reduction of portal pressure - a key factor in the pathophysiology of SFSS, though not the sole contributor.

The liver regeneration process is complex, consisting of a network of several interconnected signaling pathways that act in a coordinated manner. It is not possible to determine which pathway specifically regulates which phase of the cell cycle. In response to a stimulus (hepatectomy), the activators of these pathways (TNF-α, IL-6, HGF, EGF, and TGF-α) increase. One important pathway is the NF-κB pathway, which, once activated by TNF-α, amplifies the inflammatory response of TNF, IL-1, IL-6, and VEGF^29^ ^,^ ^45^ ^,^ ^49^.

Immunohistochemical analysis and gene expression can reflect which proteins are present and which genes are being actively transcribed, indicating cellular responses to stimuli. This allows the identification of molecules involved in the various biological processes that occur in the graft^4^ ^,^ ^36^.

Cytokines considered pro-inflammatory, such as TNF-α, IL-1, and IL-6, remain at basal levels in liver tissue and, in response to a stimulus (hepatectomy), rise rapidly, initiating the normal process of liver regeneration^23^.

In our model, the immunohistochemical expression of TNF-α did not reveal a statistically significant difference between the four times of the surgery.

TNF-α activates the transcription factor NF-κB through the tumor necrosis factor receptors 1 and 2 (TNFR-1 and TNFR-2), but the NF-κB pathway demonstrates a complex network of interactions with other pathways, such as janus kinase/signal transducer and activator of transcription (JAK/STAT), phosphoinositide 3-kinase/ protein kinase b/ mechanistic target of rapamycin (PI3K/Akt/mTOR), and canonical Wnt signaling (Wnt/β-catenin)^41^ ^,^ ^47^. These interactions may explain our findings: mild NF-κB protein expression at T0, which increases significantly at T4, even without significant changes of TNF-α. Paradoxically, gene expression did not change significantly during surgery. This may be explained by the disparity between protein and gene expression. In a previous study by our group^33^, using whole liver grafts, NF-κB levels significantly decreased after hepatic reperfusion. When compared with the current findings, it may suggest that the NF-κB pathway is involved in the pathogenesis of the syndrome.

As mentioned above, IL-6 increases in response to stimulus mainly from liver sinusoidal endothelial cells release, although the increase in IL-6 also occurs through activation of the NF-κB pathway^44^. Our findings confirm this dual regulation: IL-6 gene expression in pigs with partial grafts showed a significant increase at T4, which correlated temporally with the elevation of NF-κB protein, as described above.

Hypoxia triggers a well-orchestrated angiogenic cascade during Pringle maneuvers in hepatectomies or graft preservation in transplants. Through hypoxia-inducible factor-1α (HIF-1α), there is transcriptional activation of VEGF^26^ ^,^ ^38^. Shimizu et al. demonstrated that VEGF gene expression after hepatectomy increases 24 hours later and peaks at 48±72 hours^40^. An increase in VEGF protein in immunohistochemistry was observed from mild at T0 to moderate at T4. Longer-term studies are needed to perform measurements at other time points and determine whether this difference could be related to inadequate regeneration.

IL-10 is an important anti-inflammatory cytokine produced in the liver, which increases in response to oxidative stress, circulating catecholamines, and TNF-α, typically acting to suppress excessive inflammatory responses^45^ ^,^ ^50^. In ischemia-reperfusion studies, which involve proinflammatory cytokines, chemokines, and adhesion molecules, many of which are regulated by NF-κB, IL-10 has been shown to be hepatoprotective, inhibiting NF-κB activation in the liver after reperfusion^48^. In this model, IL-10 gene expression significantly increased at T4, which may suggest early activation of the regulatory system.

Despite the extensive literature on SFSS and liver regeneration, a significant gap remains in available experimental models. Analyzing systematic reviews by Cinelli et al.^6^ and Mohkam et al.^30^, it was observed that most small-for-size models in pigs are based on hepatectomies, which do not reproduce the preservation or ischemia-reperfusion injury and vascular dynamics of a real transplant^21^.

This work represents a methodological advance by performing the first integrated assessment of protein and gene expression of key markers (TNF-α, IL-6, IL-1, IL-10, VEGF, and NF-κB), correlated with hemodynamic (portal pressure), histopathological, and functional (graft survival) parameters.

Conducting this study presented challenges inherent to the swine model; it required a large, multidisciplinary, experienced team, advanced pre- and postoperative care protocols, and laboratories with adequate infrastructure. Despite its methodological rigor, it is acknowledged that the study had limitations: a steep learning curve for the team, even though all participants were experts in their respective fields; postoperative survival, requiring improved animal monitoring and the addition of an immunosuppression protocol; and prolonged follow-up, necessary to correlate the results obtained in this study.

The analysis of protein expression and gene expression of key mediators in liver regeneration, such as TNF-α, IL-1β, VEGF, NF-κB, IL-6, and IL-10, performed in this study, may elucidate the mechanisms involved in graft dysfunction and recovery in SFSS. The temporal characterization of these molecules may reveal the cascade of events that determine graft outcome. These findings open perspectives for future research, such as functional validation of new genetic candidates associated with liver regeneration and the development of innovative therapeutic approaches based on gene modulation, aiming to optimize the recovery of small-for-size grafts.

## CONCLUSIONS

This experimental study confirmed the technical feasibility of partial liver transplantation in a porcine model, despite the procedure’s complexity and high mortality rate. Immunohistochemical and gene expression analyses revealed intense inflammatory and immune responses during the peri- and immediate postoperative periods, characterized by a dynamic interplay between pro-inflammatory and regulatory/anti-inflammatory cytokines. These mechanisms, arising from both the body’s response to the graft and surgical trauma, offer essential insights into the pathophysiology of SFSS and support the development of targeted modulation strategies to improve outcomes.

## DATA AVAILABILITY

The datasets generated and/or analyzed during the current study are available from the corresponding author upon reasonable request.
